# Program for Healthier School Cafeterias in Rio Grande do Sul, Brazil: Protocol for a Community-Based Randomized Trial

**DOI:** 10.2196/22680

**Published:** 2021-01-19

**Authors:** Mariana Balestrin, Carla Cristina Bauermann Brasil, Ericles Andrei Bellei, Vanessa Ramos Kirsten, Mario Bernardes Wagner

**Affiliations:** 1 Faculty of Medical Sciences Federal University of Rio Grande do Sul (UFRGS) Porto Alegre Brazil; 2 Department of Foods and Nutrition Federal University of Santa Maria (UFSM) Palmeira das Missões Brazil; 3 Institute of Exact Sciences and Geosciences University of Passo Fundo (UPF) Passo Fundo Brazil

**Keywords:** school health services, healthy diet, pediatric obesity, schools, snacks

## Abstract

**Background:**

School cafeterias can promote poor eating habits, as these retail outlets have a variety of foods considered to be nonnutritive and unhealthy. However, despite the need for effective preventive strategies, there is still disagreement on the best approach due to the lack of evidence on interventions to prevent and treat obesity in the school settings.

**Objective:**

We aim to verify the efficacy of an educational intervention program to improve the hygienic conditions and the composition of the menu offered in school cafeterias in the state of Rio Grande do Sul, Brazil.

**Methods:**

We will conduct a randomized, parallel, two-arm, community-based controlled study. Elementary and high schools, both public and private, in the State of Rio Grande do Sul, Brazil, that have a cafeteria will be eligible. Schools will be recruited and randomly assigned to the intervention (n=27) or control (n=27) group. The intervention group will receive an educational intervention program based on the guidelines issued by the Ministry of Health of Brazil, consisting of a 160-hour distance-learning qualification course, for 10 weeks, and using the Moodle platform and WhatsApp app. The intervention targets the owners and people in charge of the cafeterias, food handlers, principals, vice principals, teachers, pedagogical coordinators, dietitians, representatives of students' parents, and students over 16 years old. Meanwhile, the control group will receive only a printed copy of the book containing the guidelines used. The efficacy of the intervention will be determined by the hygienic conditions of the cafeteria and the composition of the menu offered, also considering the levels of processing of food sold. All outcomes will be analyzed as intention-to-treat and per-protocol. We will use covariance analysis or a generalized linear model for continuous data and ordinal logistic regression for ordinal categorical data. The level of statistical significance considered will be P<.05 for a 95% CI.

**Results:**

This project was funded in early 2018. We administered the intervention program in 2019. All data have already been collected, and we are analyzing the data. The results are expected in 2021.

**Conclusions:**

To our knowledge, this may be the first randomized controlled study in school cafeterias held in Brazil. The results will provide evidence for the formulation of public food and nutritional security policies and for the development of effective strategies to provide safe and healthy school meals.

**Trial Registration:**

Brazilian Clinical Trials Registry RBR-9rrqhk; https://ensaiosclinicos.gov.br/rg/RBR-9rrqhk

**International Registered Report Identifier (IRRID):**

DERR1-10.2196/22680

## Introduction

Childhood obesity has been recognized as one of the greatest public health challenges of the 21st century, according to the World Health Organization [1]. By 2016, 18% of children and adolescents aged 5 to 19 years were overweight or obese worldwide [1]. Data from the Global Burden of Disease Study indicate that more than 70 countries have doubled their prevalence of obesity between 1980 and 2015, with an increase to 112 million obese children worldwide [2].

In Brazil, access to food in the school environment can occur through school meals provided by the Brazilian National School Feeding Program (PNAE - Programa Nacional de Alimentação Escolar) as well as through school cafeterias (ie, a facility within the educational establishment that aims at providing food to the school community upon payment). Data from the 2015 Brazilian National School Health Survey report that the percentages of foods considered unhealthy consumed by Brazilian students are high. The consumption of sweets, ultra-processed snacks, soft drinks, and fried snacks was 41.6%, 31.3%. 26.7%, and 13.7%, respectively [3]. The same survey revealed that these items are mostly unhealthy, nutrient-poor, and unsuitable for health promotion at school.

Studies indicate that school cafeterias end up promoting unhealthy eating habits [4,5]. A high prevalence of foods with low nutritional quality marketed in these places was identified in several observational studies in Brazil [4,6-8], as well as in other countries [9-12]. Given this scenario, evidence indicates that school interventions can have an impact on the prevention or treatment of obesity, with changes in the nutritional status and eating behavior of children and adolescents [13-17]. There is some evidence of effective interventions for better school meals; however, several systematic reviews reveal heterogeneous, low-quality studies with methodological deficiencies and very small effect sizes [12,18-23]. Moreover, few studies have addressed intervention strategies to improve the food environment of school cafeterias [10,24-27]. In addition, the maintenance of long-term effects is not yet known, and large-scale interventions can pose a considerable challenge, limiting their impact on nutrition and public health [18,26,28-30].

According to local government regulations, school food service employees must undergo food safety training [31,32]. The involvement of the entire school community in the development of these interventions is promising for the promotion of an adequate and healthy diet [33,34]. However, studies report that insufficient training appears to be a barrier to adequate food security practice [35]. Review studies suggest that food safety education training is effective for improving the knowledge of food handlers, but more evidence is needed to improve behavior change [36,37].

To the best of our knowledge, there has been no randomized controlled trial conducted in school cafeterias in Brazil. Addressing this gap, we have been developing an educational intervention program with a multicomponent strategy to improve the hygienic conditions and composition of the menu offered in school cafeterias in the state of Rio Grande do Sul, Brazil.

## Methods

This protocol is reported in accordance with the statement of the Consolidated Standards of Reporting Trials (CONSORT) [38] and Standard Protocol Items: Recommendations for Interventional Trials (SPIRIT) [39]. We will conduct a randomized, parallel, two-arm, community-based controlled study in school cafeterias in the state of Rio Grande do Sul, Brazil. The Research Ethics Committee of the Federal University of Rio Grande do Sul (report 89504618.9.0000.5347) approved this project, which was registered in the Brazilian Platform of Clinical Trials under the code RBR-9rrqhk on April 30, 2018 (Universal Trial Number U1111-1213-1614).

### Recruitment and Eligibility Criteria

A total of 330 schools will be assessed for eligibility to participate in the study. The definition of these schools will be obtained through data from the Education Department of the State of Rio Grande do Sul [40]. Confirmation or updates of the existing data will be verified by phone calls to all schools. We will invite to participate elementary and high schools of the public and private sectors located in the Cidadania Noroeste Colonial region, State of Rio Grande do Sul, Brazil, which total 36 municipalities according to the last official census [41].

Schools will be eligible to participate if they have a school cafeteria and they show interest in participating in the research upon signature of a consent form. Schools with exclusive care for children with special needs will be excluded, as they require a different standard of care. Schools will not be excluded based on other characteristics such as size, socioeconomic indicators, and others. Eligibility criteria will be applied prior to randomization.

Eligible schools will be personally invited to participate in the study by the research team. This strategy will be used to ensure the desired sample size. The school principal will sign a consent form for participation. In the case of outsourced cafeterias, we will request the consent of its owner. Each school will nominate its representatives to participate in the study. The principal will be responsible for obtaining the consent of the nominated members.

### Sampling

The sample size calculation was based on primary outcomes. For a significance level of 5% and statistical power of 90% to detect an effect magnitude (*d*) of 0.90, 54 schools will be needed, considering 27 for the intervention group and 27 for the control group. We considered a 10% sample loss to estimate an increase in the sample size. The estimated number of participants required to achieve the study objectives was also based on the study by Nathan et al [24], which aimed at examining whether a theoretically conceived multicomponent intervention was effective in enhancing the implementation of a healthy cafeteria policy in Australian primary schools. The sample size calculation was performed using the Power and Sample Size software (HyLown Consulting LLC, Atlanta, GA).

### Randomization and Allocation

Randomization will use a minimization process [42] to balance the number of schools between the 2 groups. This approach ensures an excellent balance between groups for several prognostic factors, even in small samples. According to Egbewale [43], the minimization process makes the evaluated groups similar in important characteristics, mainly in trials that involve a small sample and have several prognostic factors to be balanced [38,44]. This procedure decreases the chances of significant discrepancies in baseline prognostic factors that may occur at random [45]. Minimization also controls the imbalances in baseline variables more efficiently than simple randomization, since, in small samples, simple randomization can produce a biased and misleading effect [43,46].

To maintain the balance between groups and prevent disproportionate distribution from occurring, 4 predictors of interest for the allocation will be considered: city, cafeteria administration (school-owned versus outsourced), school scope (public versus private), and the number of students (<500 students versus ≥500 students). An independent researcher will perform the randomization by minimization using a computer-generated technique in SPSS 26 (IBM Corp, Armonk, NY), avoiding possible influence on the allocation. The randomization unit will be schools with cafeterias.

**Figure 1:**
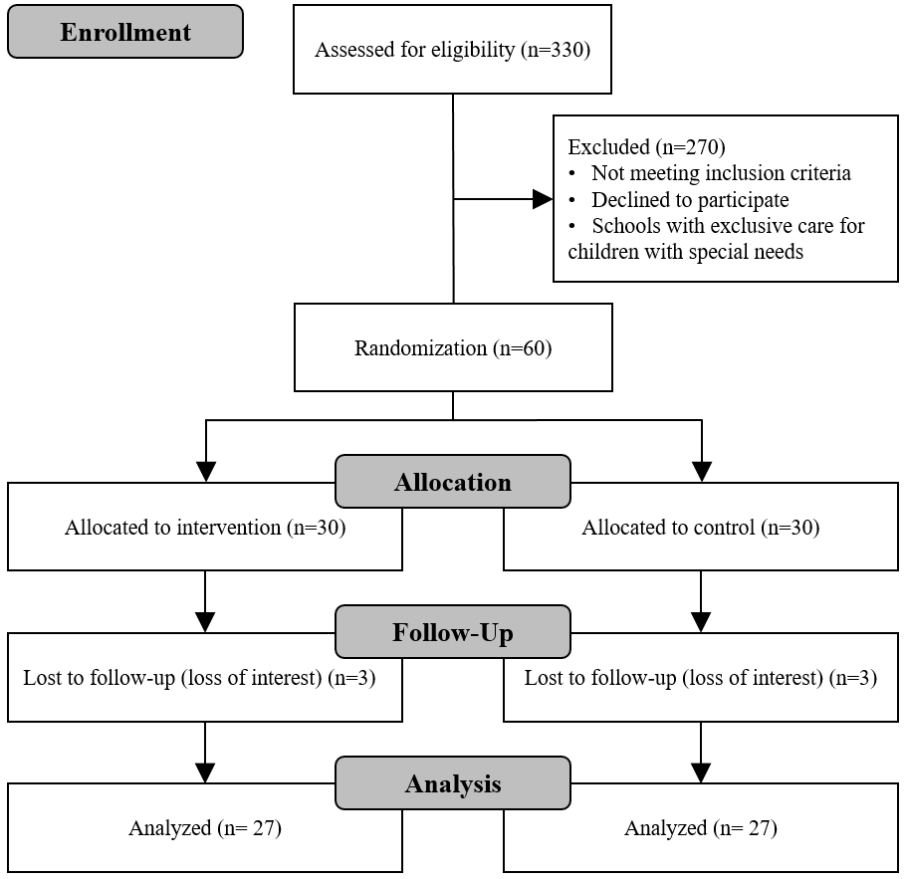
Planned study flowchart.

As it will be an educational intervention, the researchers in charge of administering the interventions and assessing the outcomes will not be able to be blinded. Therefore, to avoid interference between the control and intervention groups, blindness will be arranged for the statistician in charge of data randomization and analysis. Participant flow during each stage of the study can be seen in the estimated study flowchart in Figure 1.

### Intervention Group

After completing randomization at baseline, schools located in the intervention group will be invited to participate in the “Healthy Cafeteria: we support this idea!” Program (*Cantina Saudável: a gente apoia essa ideia!* originally in Brazilian Portuguese). This is an educational intervention consisting of a 160-hour asynchronous distance-learning qualification course using the Moodle platform. Each school from the intervention group will also have a support group on WhatsApp (Facebook Inc, Menlo Park, CA), including all the participants enrolled by the school. The course will have a duration of 10 weeks, with a workload of 16 hours per week.

The target audience of the intervention will be the owners and people in charge of the cafeterias, food handlers, principals, vice principals, teachers, pedagogical coordinators, dietitians, representatives of students’ parents, and students over 16 years old. Each week, a course module will be available on a distance-learning platform through lectures, texts, videos, and activities. Moreover, a discussion forum will be developed for each module to encourage learning, share experiences, provide support, and instigate community interaction. In each module, we will provide practical activities to be held in school cafeterias. Our teaching strategy will implement context-based and problem-solving activities. The modules will be made available in stages, so that the workload is fulfilled according to the plan. In addition, the modules will be available until the end of the course, and it is not necessary to meet fixed hours and deadlines for most course activities. This flexibility of distance learning allows participants to adapt their studies according to their routine.

The principal of each school in the intervention group will receive 2 phone calls during the intervention: the first one in the 4th week and the other in the 7th week. Both calls will be for support and follow-up for the intervention program. Calls will last approximately 5 minutes, and we will ask the director if their participants need any instructions or assistance. In addition, participants will be able to chat with a tutor throughout the intervention via chat using the Moodle platform and WhatsApp. These strategies will be used to avoid participant drop-outs.

The material used as a basis for developing the education intervention will be the book “Manual das cantinas escolares saudáveis: promovendo a alimentação saudável” (Guide of the Healthy School Cafeteria: Promoting the Healthy Food), developed by the Ministry of Health of Brazil [47]. This document guides the implementation of the school cafeterias throughout Brazil. The material was adapted according to the characteristics of the region of Brazil where the courses will be developed. The course will consist of 8 modules, depicted in Textbox 1.

**Components of the intervention program for a healthier school cafeteria.**

Module 1 - Starting the healthy school cafeteria

- Goals
  To present relevant information about the health of children and adolescents
  To study how schools and cafeterias can promote healthy and proper eating
  To understand the importance of implementing healthy school cafeterias and reasons to change
  To get to know the current laws that provide for regulation in the supply of food in school cafeterias
- Intervention items and components
  Expository classes, using slides, with an introduction of the theme and concepts for implementing a healthy cafeteria in the school setting
  Current legislation regulating the supply of food in school cafeterias in Brazil
  A video with a report on the law of cafeterias in southern Brazil
  Hands-on activity: participants are instructed to check what is sold at school

Module 2 - What is healthy eating?

- Goals
  To get to know the concept of adequate and healthy eating
  To learn what is healthy eating based on the Brazilian Population Food Guide
- Intervention items and components
  Expository class, using slides, on the importance of healthy eating in schools and the concept of adequate and healthy eating
  Videos about the concept of healthy eating
  Indication of a film about the childhood obesity epidemic
  Hands-on activities: participants are instructed to classify food sold in the cafeteria based on the concepts learned, create a campaign to encourage the consumption of healthy foods in school (eg, “buy 10 healthy items, get the 11th item for free”; “collect and exchange your points for products”). In addition, teachers are instructed on how to apply the concepts of healthy eating in the classroom

Module 3 - Cafeteria and industrialized foods

- Goals
  To get to know the effects of the consumption of industrialized foods on the student's health
  To learn how to choose foods by reading labels and nutritional information
- Intervention items and components
  Expository class, using slides, on industrialized foods and their effects, involving the reading of food labels
  Educational videos that teach the participant to choose healthy foods by reading the labels
  Suggesting an app to aid in healthy choices: Desrotulando
  Hands-on activity: participants are instructed to remove from the cafeteria foods they consider unhealthy

Module 4 - Healthy snacks

- Goals
  To provide suggestions for healthy and creative snacks to be offered in school cafeterias
- Intervention items and components
  Expository class, using slides, with suggestions for healthy and creative snacks
  A video about healthy snacks at school
  eBook with healthy recipes for school cafeterias
  Hands-on activity: participants are instructed to develop a menu with healthy snack options for the cafeteria.

Module 5 - Food hygiene

- Goals
  To learn about the importance of adopting good practices in food handling to ensure the sanitary quality and safety of food sold in the school cafeteria
- Intervention items and components
  Expository class, using slides, about food hygiene
  Videos on food handler hygiene and the correct way to sanitize vegetables and fruits
  Hands-on activities: participants are instructed to record a video sanitizing their hands, take a selfie wearing a hygiene cap, and draw up a poster to prohibit unauthorized people from entering the cafeteria.

Module 6 - Food and nutrition education

- Goals
  To present educational strategies and activities to promote adequate and healthy eating in the school setting
- Intervention items and components
  Expository class, using slides, about food and nutrition education activities
  A video addressing the importance of promoting adequate and healthy eating in the school curriculum
  Hands-on activity: participants are encouraged to develop a food and nutrition education activity based on the content learned.

Module 7 - How to profit from healthy school cafeterias and successful experiences

- Goals
  To present strategies on how to profit from the sale of healthy foods and show successful experiences in planning and implementing the healthy school cafeteria
- Intervention items and components
  Expository class, using slides, on strategies to profit from healthy school cafeterias
  Videos showing successful experiences in the implementation of a healthy school cafeteria
  Hands-on activity: participants are instructed to create a healthy recipe with a creative name and disseminate it to the school community.

Module 8 - Schedule of activities and how to keep the school cafeteria healthy

- Goals
  To propose a schedule of actions that must be carried out for the school to implant and maintain a healthy school cafeteria
- Intervention items and components
  Expository class, using Microsoft PowerPoint, with actions that must be performed in school for the implementation and maintenance of the healthy school cafeteria
  Hands-on activity: participants are encouraged to reflect on the changes made in the cafeteria (positive and negative points) during the intervention, including what still needs to be improved and what goals will be set to keep the school cafeteria healthy.

All components of the program will be offered to all participants in the school community because the content of the program does not require specific knowledge or prior training, meeting the requirements of the book on which it was based [47]. No payment nor refund will be made for cafeterias and schools to participate in the study. Local law does not allow individuals to participate in research for remuneration [48]. Schools that agree to participate will be aware of the need for the participants to dedicate time and adapt their work routine during the intervention period. Although the course includes 160 hours, this number of hours does not refer only to attending classes, but rather considers engagement in all activities necessary for the intervention process, such as talking to the school staff and performing the proposed tasks and assignments. At baseline and after follow-up, cafeterias will receive feedback on their performance, and the situation will be assessed.

There was a post hoc change in the protocol of this study in relation to what was originally planned in the trial registry. Initially, we stipulated that the intervention program would be delivered in 160 hours over 20 weeks, therefore, with a workload of 8 hours per week. It was also foreseen that the course would have 140 hours in distance mode and 20 hours of in-person mode. However, in new planning, we defined that the program would be delivered only in the distance mode and in a shorter period of time, with a weekly workload of 16 hours over 10 weeks. We justify this because of the limited funding available and the considerable geographical distance between the locations of participating schools.

### Control Group

The control group will not receive any type of active intervention. After data collection is completed at baseline, schools from the control group will receive a printed copy of the book “Guide of the Healthy School Cafeteria: Promoting the Healthy Food” developed by the Ministry of Health of Brazil [47]. At baseline and after follow-up, cafeterias will receive feedback on their performance, and the situation will be assessed.

### Data Collection and Management

**Figure 2:**
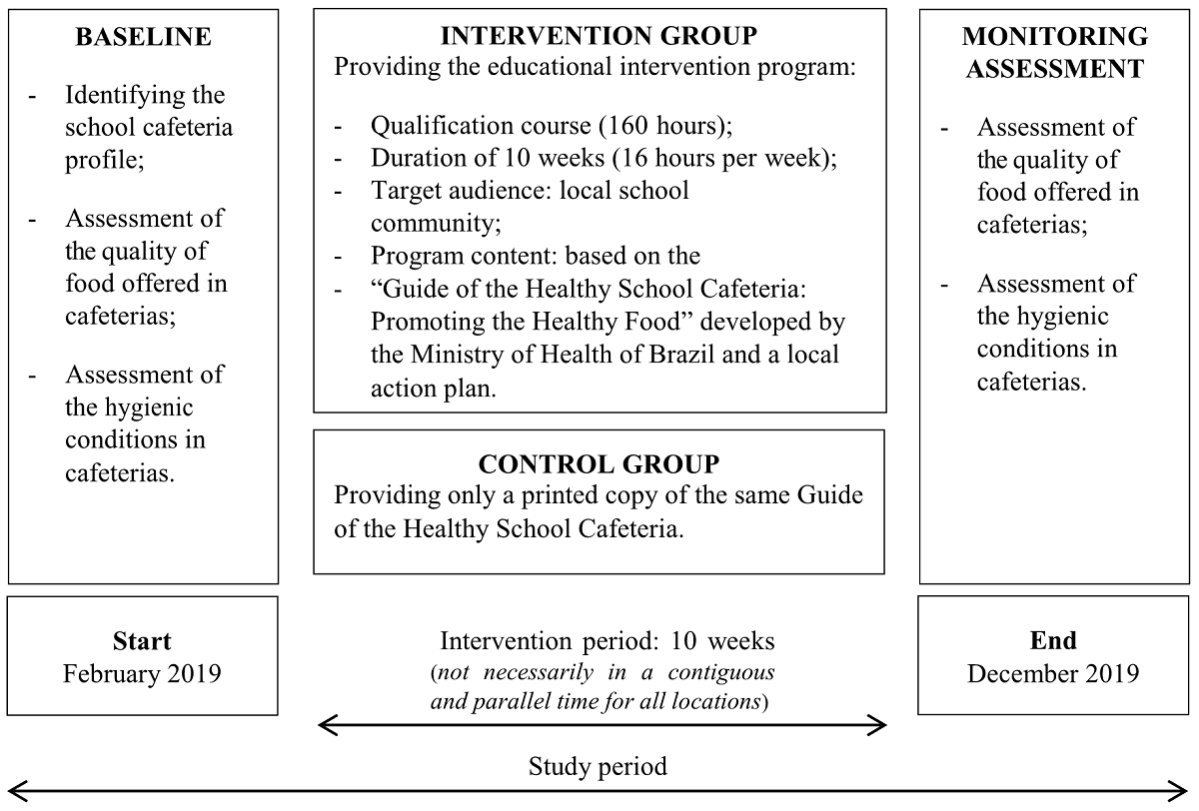
Study overview.

The study will be conducted in 3 stages, depicted in Figure 2. Data to assess outcomes will be collected at baseline and after follow-up. Not all interventions will be carried out in a contiguous and parallel time for all locations since the schools to be selected may be very distant geographically, and this will require more effort and time from researchers.

Researchers responsible for collecting data on site will be previously trained and will receive a guidance manual with information on the collection order, data completeness, researcher’s characterization and identification, behavior, actions, language, and guidelines on the observation of hygienic conditions. The investigation of the profile of school cafeterias will be carried out through a cross-sectional assessment using a printed questionnaire with the owners or people in charge of the school cafeterias and the school directors. This characterization of the study population will be done at the beginning of the study, simultaneously with the data collection of baseline variables, and it will be carried out before randomization by the researcher tasked with the collection of outcome measures. The data on the profile of the school and the cafeteria will be collected through a face-to-face interview with the principal and head of the cafeteria, respectively, using a printed demographic questionnaire.

The questionnaire for collecting demographic data will be based on studies by Giacomelli [49] and Porto et al [50]. The following information will be included: characterization of the school, type of management, educational stage, number of students, presence of school meals, type of cafeteria management, number of people working in the cafeteria, opening hours, number of snacks served, place of production of snacks, aspects involved with the choice of food offered, presence of other types of sale within the school, and presence of a dietitian. The questions related to the composition of the menu offered in school cafeterias according to the level of food processing will be based on the recommendations of the Food Guide for the Brazilian Population [51] and on the studies by Wolfenden et al [52] and Williams et al [25]. Meanwhile, the questions related to hygienic conditions will be obtained from the assessment frameworks already developed and validated by the Brazilian Health Regulatory Agency, which is equivalent to the US Food and Drug Administration [31,53]. Data entry from all instruments will be performed in parallel and with redundant copies to verify the integrity and concordance of collected data.

We will analyze the fidelity and dose of the intervention based on the reports of participants' usage of the Moodle platform. This includes access logs with the day and time that the participant accessed the platform, all actions performed, list of activities and assignments, number and history of views, the fulfillment of activities, dwell time, and other information. In addition, participation will be verified based on the history of submitted assignments and participation in the discussion forums. The assessment of the feasibility and acceptance of the intervention will be measured based on the changing effects of the assessed outcomes, usage reports of Moodle, and an assessment of the participants' acceptance and their perception of the course, through an online retrospective questionnaire (Multimedia Appendix 1) applied at the end of the program.

### Outcome Measures

We aim to verify the efficacy of an educational intervention program to improve the hygienic conditions and the composition of the menu offered in school cafeterias in the state of Rio Grande do Sul, Brazil. The efficacy of the intervention will consider 2 primary outcomes: (1) composition of the menu offered (percentages of fresh, processed, and ultraprocessed foods) and (2) assessment of hygienic conditions concerning good practices in handling food. The secondary outcome will be a score calculated according to the level of processing of food sold. These variables will be collected at baseline and immediately after the intervention.

#### Primary Outcomes

The assessment of the impact of the intervention on the composition of the menu offered in school cafeterias will be based on similar Australian studies [25,54]. The assessment of the school cafeteria menu composition will be determined by counting all items sold and the percentages of food classified according to the level of industrial processing proposed by the Food Guide for the Brazilian Population [51] and Monteiro et al [55]. A modified version of these references was used for this research. Group 1 (F*fresh*) includes fresh foods, minimally processed and culinary preparations without the addition of culinary ingredients (salt, sugar, oils, fats, or other ingredients). Group 2 (F*process*) includes processed foods and culinary preparations with culinary ingredients. Group 3 (F*ultra*) includes ultraprocessed foods. The culinary ingredients group proposed by Brazil [31] was excluded from the classification because the cafeterias do not sell these foods in isolation. Two properly trained dietitians will carry out this assessment using pen and paper. In case of divergence, a third dietitian will be consulted.

Good practices in food handling are a set of procedures that food services implement to ensure food quality to the consumer, minimizing possible harm to health, especially that caused by foodborne diseases. In this sense, there is a need to assess and classify the food sold according to the characteristics that include the amount of microbial and chemical contamination [56]. To collect data regarding hygienic conditions, we will use an instrument that has been validated by the National Health Surveillance Agency [57], which is based on Brazilian legislation, Regulatory Ordinance 817, published on May 10, 2013, by the Ministry of Health [56]. This legislation is composed of 51 items from 9 categories: water supply; building structure; cleaning of facilities, equipment, furniture, and utensils; control of vectors and urban pests; food handlers; raw material, ingredients, and packaging; food preparation; storage, transport, and display of prepared food; and liability, documentation, and registration.

The assessment of the hygienic conditions of the cafeterias consists of a continuous scoring system that ranges from 0 (less severe) to 2498.89 (more severe). The score will be assigned when the evaluated cafeteria does not meet some of the requested requirements, so the higher the score, the greater the number of nonconformities verified and the worse the performance of the establishment [58]. The scores for each of the 51 items checked will be defined based on risk criteria, in order to identify those that have the most direct impact on the quality of food and on the health of consumers. In the score, the Impact Index will be used, representing the importance in the prevention of foodborne diseases, as well as the Factor Load of the items, as validated by the National Health Surveillance Agency [57]. This assessment will take place through an onsite inspection carried out by a trained dietitian using a printed checklist.

#### Secondary Outcome

To assess the impact of the intervention on the food sold in the cafeteria, we have prepared a score that can be calculated based on the frequency of food available for sale and its classification according to the level of industrial processing proposed by Brazil [51] and Monteiro et al [55]. This score was developed due to the lack of a standard method for analyzing food in the school environment [59] and was also based on similar Australian studies [25,26]. Therefore, in addition to evaluating the composition of the menu offered in primary outcomes, we also chose to develop an equation that uses the frequency of each type of food but makes these variables continuous for a secondary outcome.

After data collection, all types of food and drinks sold in the cafeterias will be classified according to their level of processing. In the second stage, for each cafeteria, the frequency of items available in each category will be counted, multiplied by the standardized weight for the Group of fresh foods, Group of processed foods, and Group of ultraprocessed foods, which are +1, 0, and -1, respectively, as they are healthy, neutral, and unhealthy, respectively.

Equation (1) is used to calculate the score of the cafeteria for the level of food processing, where F*fresh* = frequency of fresh food items; F*process* = frequency of processed foods; F*ultra* = frequency of ultraprocessed foods; n = total number of items sold in the cafeteria, achieved by multiplying the frequency of food in each category by its corresponding weight, added to the values and dividing by the total number of items sold in the cafeteria. The score formula 50R + 50 is used to obtain scores for each establishment.


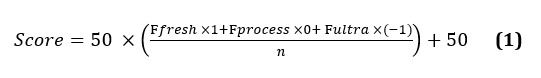


The score can vary on a scale of 0 to 100 points, where 50 is the midpoint. Thus, a cafeteria that offers healthy (fresh) and unhealthy (ultraprocessed) food in equal quantities will receive an average score of 50 points and will be considered a neutral cafeteria. Cafeterias that reach a score below 50 points have a greater predominance of ultraprocessed foods (ie, a greater offering of unhealthy foods). In summary, higher scores reflect better food quality in the school cafeterias.

### Statistical Analysis

We will consider the 2 primary outcomes and the secondary outcome: composition of the menu offered (ordinal categorical data), assessment of hygienic conditions concerning good practices in handling food (continuous variables), and the score for the level of processing of food sold (continuous variables). The baseline characteristics of the school and the cafeteria will be presented in conventional descriptive statistics. Continuous variables will be presented as means and standard deviations for symmetric data or medians and interquartile ranges for asymmetric data, while categorical variables will be presented as frequencies and percentages. All outcomes will be analyzed as intention-to-treat and per-protocol. The intention-to-treat analysis will include all participants according to their original group assignment, regardless of what happened later. The per-protocol analysis will consider only those participants who comply with the protocol in terms of eligibility, interventions, and evaluation of results [38]. Unadjusted and adjusted estimates allowing for the potential confounding effects of all minimization factors will be presented for primary and secondary endpoints. Multiple imputation will be used to deal with missing data [60].

For the analysis of continuous data (hygienic conditions and level of processing of food sold), analysis of covariance or a generalized linear model with an appropriate link function will be used, depending on the data characteristics. The outcomes will be assessed by comparing the mean changes (delta) between baseline and follow-up values between the groups, adjusting for the baseline value of the outcome. For the analysis of ordinal categorical data (menu composition and percentages of each type of food), we will use an ordinal logistic regression model to assess between-group differences at follow-up, adjusting for the baseline value of the outcome. All data will be analyzed using the statistical software SPSS 26.0 (IBM Corp, Armonk, NY) and R Software 3.5.0 (R Development Core Team). The statistical level of significance considered will be *P*<.05 for a confidence interval of 95%.

## Results

This project started receiving funds in early 2018. The educational program was developed in 2018. We administered the intervention program in 2019. All data have already been collected, and we are analyzing the data. In 2020, to ensure ethical and opportunity principles, we offered the educational program to schools from the control group that expressed interest after completion of follow-up. The results are expected in 2021.

## Discussion

This protocol presents a description of the methods to be used to assess the effect of an intervention for the implementation of healthy school cafeterias, ensuring the accuracy of the study and allowing its reproducibility. To our knowledge, this may be the first randomized controlled study in school cafeterias held in Brazil. After the completion of this study, it will be possible to determine whether the proposed educational intervention program is able to improve the menu offered and hygienic conditions of school cafeterias. The results will provide evidence for the formulation of public nutritional and food security policies and for the development of efficacy strategies to provide safe and healthy school meals.

## Appendix

Multimedia Appendix 1 Retrospective questionnaire applied at the end of the intervention program.

Multimedia Appendix 2 Peer review report from the grant agency.
