# Supplementary material for: Program for Healthier School Cafeterias in Rio Grande do Sul, Brazil: Protocol for a Community-Based Randomized Trial
Source: JMIR Res Protoc. 2021 Jan 19;10(1):e22680. doi: 10.2196/22680 (PMC7854040; doi:10.2196/22680)
Supplement: Multimedia Appendix 1 [file resprot_v10i1e22680_app1.pdf]

Formulário de satisfação com o curso

Olá,
Agradecemos por você ter efetuado o Curso de Educação a Distância Cantina Saudável: a gente apoia essa ideia.
Por meio desse breve questionário, sem necessidade de identificar a sua identidade, almejamos avaliar os aspectos gerais em relação a realização do curso.

1) Eu realizei o curso utilizando a plataforma:

- ◯ Moodle
- ◯ WhatsApp
- ◯ Ambas principalmente o Moodle
- ◯ Ambas principalmente o WhatsApp

2) O emprego das ferramentas Moodle e WhatsApp foi proveitoso?

- ◯ Discordo totalmente
- ◯ Discordo em parte
- ◯ Indiferente
- ◯ Concordo em parte
- ◯ Concordo totalmente

3) Em relação a questão número 2 faça comentários sobre a sua resposta:

Sua resposta

4) Como você realizou o curso?

- ◯ Em grupo na escola, conforme o sugerido
- ◯ Em grupo, porém cada um fez a leitura sozinho
- ◯ Sozinho

5) Justifique a sua resposta em relação a questão quatro. (Como foi o aproveitamento da atividade em grupo ou sozinho)

Sua resposta

6) Com qual recurso/técnica de ensino você aprendeu melhor?

- ◯ Apresentação em PDF
- ◯ Imagens
- ◯ Mensagens por e-mail
- ◯ Fórum
- ◯ Mensagens por WhatsApp
- ◯ Atividades e Tarefas
- ◯ Vídeos
- ◯ Outro: ______

7) Você fez o uso ou a leitura das cartilhas, materiais e vídeos enviados como materiais complementares?

- Sim
- Não
- Não Sei

8) O plano de ensino do curso foi seguido.

- Discordo totalmente
- Discordo em parte
- Indiferente
- Concordo em parte
- Concordo totalmente
- Não sei responder

9) Os tutores sanaram dúvidas sobre o conteúdo ministrado e as ferramentas de aprendizagem

- Discordo totalmente
- Discordo em parte
- Indiferente
- Concordo em parte
- Concordo totalmente

10) Como você avalia a sua aprendizagem e o entendimento dos conteúdos tratados ?

Sua resposta

11) A comunidade escolar se envolveu e apoiou na transformação da cantina de sua escola em um ambiente mais saudável?

- Discordo totalmente
- discordo em parte
- indiferente
- concordo em parte
- concordo totalmente

12) Como você observou a receptividade dos demais estudantes em relação as mudanças na cantina propostas pelo curso?

- Não apoiaram
- indiferentes
- Apoiaram parcialmente
- Apoiaram totalmente

13)Qual era o seu objetivo ao realizar o Curso Cantina Saudável? (é possível marcar mais de uma opção)

- [ ] Adequar a cantina a nova lei estadual
- [ ] Avaliar o que pode ser melhorado na cantina da escola
- [ ] Aprender sobre a higiene correta dos alimentos, mãos e ambiente
- [ ] Aprender sobre alimentação saudável na escola
- [ ] Outro(s) (aberto)

14) Aprendi e compreendi os seguintes assuntos tratados. (mais de uma opção):

- [ ] Nenhum
- [ ] O que é alimentação saudável
- [ ] Higiene dos alimentos, ambiente e manipulador
- [ ] Como tornar a cantina mais saudável
- [ ] Adequação a cantina a nº15.216/2018
- [ ] Como lucrar
- [ ] Opções de lanches mais saudáveis
- [ ] Tornar a cantina escolar um ambiente promotor da alimentação saudável
- [ ] Atividades de educação alimentar e nutricional
- [ ] Todos

15)Qual foi o tema/item que você conseguiu colocar (ou mudar) em prática na cantina ou na escola?

- ◯ Nenhum
- ◯ O que é alimentação saudável
- ◯ Higiene dos alimentos, ambiente e manipulador
- ◯ Como tornar a cantina mais saudável
- ◯ Adequação a cantina a lei nº15.216/2018
- ◯ Como lucrar
- ◯ Opções de lanches mais saudáveis
- ◯ Tornar a cantina escolar um ambiente promotor da alimentação saudável
- ◯ Atividades de educação alimentar e nutricional
- ◯ Opção 10

16) O curso atendeu as suas expectativas?

Sua resposta

17)Você gostaria de compartilhar alguma opinião crítica, elogio e sugestão para aprimorar o curso?

Sua resposta

Obrigada por reservar um tempo e pela dedicação na realização do Curso de Educação a Distância Cantina Saudável: a gente apoia essa ideia!

Enviar

Nunca envie senhas pelo Formulários Google.

Este conteúdo não foi criado nem aprovado pelo Google. Denunciar abuso - Termos de Serviço - Política de Privacidade

Google Formulários
