# Supplementary material for: Program for Healthier School Cafeterias in Rio Grande do Sul, Brazil: Protocol for a Community-Based Randomized Trial
Source: JMIR Res Protoc. 2021 Jan 19;10(1):e22680. doi: 10.2196/22680 (PMC7854040; doi:10.2196/22680)
Supplement: Multimedia Appendix 2 [file resprot_v10i1e22680_app2.pdf]

## Resultado Final

### Identificação da Proposta

**Número do Processo:** 442730/2016-0 **Solicitante:** Vanessa Ramos Kirsten

**Chamada:** UNASUL Fase II

**Título do Projeto:** SEGURANÇA ALIMENTAR EM CANTINAS ESCOLARES DOS MUNICIPÍOS DO TERRITÓRIO DA CIDADANIA DO NOROESTE COLONIAL/RS: DIAGNÓSTICO E INTERVENÇÃO

### Parecer de Deliberação Final

**Nota Final**

| Nota | Ordem | Prioridade |
|---|---|---|
| 8,81 | | P001 |

**Resultado da Avaliação**

**Favorável**

**Justificativa:**

A proposta apresenta tema relevante na área da educação alimentar e nutricional, com ênfase em educação escolar e consumo saudável, bem contextualizada. Os objetivos são claros, com metodologia compatível e cronograma adequado. O CL do proponente revela experiência relativa na área, destacando-se, necessidade de maior atuação na formação de recursos humanos, para que possam repercutir positivamente em futuras contribuições acadêmicas e com os atores do segmento da SAN, contudo visualiza-se potencial para o gerenciamento do mesmo, juntamente com a equipe composta. Recomenda-se ajuste no orçamento.

**Recursos**

| Capital | Custeio | Bolsa | Valor Total |
|---|---|---|---|
| R$ 9.200,00 | R$ 20.000,00 | R$ 21.840,00 | R$ 51.040,00 |

**Data de Emissão**

**Data de Emissão do Parecer:** 18/09/2017

**Parecer de Deliberação final antes do período recursal**

**Critério: Adequação da metodologia proposta**

**Peso:** 1.0 **Nota:** 9.0

**Critério: Aderência ao objeto e diretrizes do edital (subitens 1.1 e 1.1.2).**

**Peso:** 2.0 **Nota:** 9.0

**Critério: Adequação e compatibilidade do orçamento aos objetivos, atividades e metas propostos.**

**Peso:** 1.0 **Nota:** 9.0

**Critério: Mérito, originalidade e relevância da proposta para o desenvolvimento científico, tecnológico e de inovação do País dentro dos objetivos da Chamada**

**Peso:** 2.0 **Nota:** 9.0

**Critério: Constituição de parceria(s) formalizada(s) entre instituições brasileiras e obrigatoriamente com pelo menos uma instituição da UNASUL que atue em atividades de SAN**

**Peso:** 1.0 **Nota:** 8.0

**Critério: Potencial de aplicabilidade e contribuição da proposta para o desenvolvimento sustentável local/regional, possibilitando ao público beneficiário prioritário a construção e socialização de conhecimentos, tecnologias e metodologias**

**Peso:** 2.0 **Nota:** 9.0

**Critério: Atendimento ao Público Beneficiário Prioritário definido no item 1.3**

**Peso:** 1.0 **Nota:** 9.0

**Critério: Experiência prévia do proponente na área da proposta assim como sua capacidade técnica e gerencial para a execução do objeto**

**Peso:** 1.0 **Nota:** 8.0

**Critério: Adequação do cronograma de entrega dos produtos esperados como resultado do projeto**

**Peso:** 1.0 **Nota:** 9.0

**Critério: Coerência e adequação entre a capacitação e a experiência do coordenador e da equipe do projeto aos objetivos, atividades e metas proposta**

**Peso:** 1.0 **Nota:** 8.5

**Nota Final**

| Nota | Ordem | Prioridade |
|---|---|---|

**Resultado da Avaliação**

**Favorável**

**Justificativa:**

A proposta apresenta tema relevante na área da educação alimentar e nutricional, com ênfase em educação escolar e consumo saudável, bem contextualizada. Os objetivos são claros, com metodologia compatível e cronograma adequado. O CL do proponente revela experiência relativa na área, destacando-se, necessidade de maior atuação na formação de recursos humanos, para que possam repercutir positivamente em futuras contribuições acadêmicas e com os atores do segmento da SAN, contudo visualiza-se potencial para o gerenciamento do mesmo, juntamente com a equipe composta. Recomenda-se ajuste no orçamento.

**Recursos**

| Capital | Custeio | Bolsa | Valor Total |
|---|---|---|---|
| R$ 9.200,00 | R$ 20.000,00 | R$ 21.840,00 | R$ 51.040,00 |

**Data de Emissão**

**Data de Emissão do Parecer:** 28/07/2017

**Parecer de Recomendação**

**Critério: Adequação da metodologia proposta**

**Peso:** 1.0 **Nota:** 9.0

**Critério: Aderência ao objeto e diretrizes do edital (subitens 1.1 e 1.1.2).**

**Peso:** 2.0 **Nota:** 9.0

**Critério: Adequação e compatibilidade do orçamento aos objetivos, atividades e metas propostos.**

**Peso:** 1.0 **Nota:** 9.0

**Critério: Mérito, originalidade e relevância da proposta para o desenvolvimento científico, tecnológico e de inovação do País dentro dos objetivos da Chamada**

**Peso:** 2.0 **Nota:** 9.0

**Critério: Constituição de parceria(s) formalizada(s) entre instituições brasileiras e obrigatoriamente com pelo menos uma instituição da UNASUL que atue em atividades de SAN**

**Peso:** 1.0 **Nota:** 8.0

**Critério: Potencial de aplicabilidade e contribuição da proposta para o desenvolvimento sustentável local/regional, possibilitando ao público beneficiário prioritário a construção e socialização de conhecimentos, tecnologias e metodologias**

**Peso:** 2.0 **Nota:** 9.0

**Critério: Atendimento ao Público Beneficiário Prioritário definido no item 1.3**

**Peso:** 1.0 **Nota:** 9.0

**Critério: Experiência prévia do proponente na área da proposta assim como sua capacidade técnica e gerencial para a execução do objeto**

**Peso:** 1.0 **Nota:** 8.0

**Critério: Adequação do cronograma de entrega dos produtos esperados como resultado do projeto**

**Peso:** 1.0 **Nota:** 9.0

**Critério: Coerência e adequação entre a capacitação e a experiência do coordenador e da equipe do projeto aos objetivos, atividades e metas proposta**

**Peso:** 1.0 **Nota:** 8.5

**Nota Final**

| Nota | Ordem | Prioridade |
|---|---|---|

**Resultado da Avaliação**

**Recomendada**

**Justificativa:**

A proposta apresenta tema relevante na área da educação alimentar e nutricional, com ênfase em educação escolar e consumo saudável, bem contextualizada. Os objetivos são claros, com metodologia compatível e cronograma adequado. O CL do proponente revela experiência relativa na área, destacando-se, necessidade de maior atuação na formação de recursos humanos, para que possam repercutir positivamente em futuras contribuições acadêmicas e com os atores do segmento da SAN, contudo visualiza-se potencial para o gerenciamento do mesmo, juntamente com a equipe composta. Recomenda-se ajuste no orçamento.

**Recursos**

| Capital | Custeio | Bolsa | Valor Total |
|---|---|---|---|
| R$ 9.200,00 | R$ 20.000,00 | R$ 21.840,00 | R$ 51.040,00 |

**Data de Emissão**

**Data de Emissão do Parecer:** 31/03/2017

**Parecer de Pré-seleção**

**Critério: O proponente do projeto possui título de Mestre/Doutor (subitem 3.2 - b)?**

SIM

**Critério: O coordenador do projeto possui vínculo empregatício com a Instituição de execução do projeto (subitem 3.2 - d)?**

SIM

**Critério: Foram solicitados itens não financiáveis pelo projeto, de acordo com o subitem 5?**

NÃO

**Critério: A instituição de execução do projeto obedece ao disposto no subitem 3.3?**

SIM

**Nota Final**

| Nota | Ordem | Prioridade |
|---|---|---|

**Resultado da Avaliação**

Enquadrado

**Justificativa:**

A proposta atende aos critérios de elegibilidade estabelecidos pela Chamada CNPq/MCTIC Nº 016/2016 (item 3).

**Data de Emissão**

**Data de Emissão do Parecer:** 15/02/2017

CNPq
Conselho Nacional de Desenvolvimento Científico e Tecnológico

2786184626776925

# TERMO DE ACEITAÇÃO DE APOIO FINANCEIRO A PROPOSTA DE NATUREZA CIENTÍFICA, TECNOLÓGICA E/OU DE INOVAÇÃO

Processo: 442730/2016-0
Titulo do Projeto: SEGURANÇA ALIMENTAR EM CANTINAS ESCOLARES DOS MUNICIPÍOS DO TERRITÓRIO DA CIDADANIA DO NOROESTE COLONIAL/RS: DIAGNÓSTICO E INTERVENÇÃO
Instituição de Vínculo: Universidade Federal de Santa Maria/UFSM-RS
CNPJ: 95591764000105
Instituição de Execução: Universidade Federal de Santa Maria
CNPJ: 95591764000105
Chamada: Chamada CNPq/MCTIC Nº 016/2016 - SEGURANÇA ALIMENTAR E NUTRICIONAL NO ÂMBITO DA UNASUL
Eu, Vanessa Ramos Kirsten , 000.260.470-12, declaro conhecer, concordar e atender integralmente às exigências Nº CPF (ou PASSAPORTE, se estrangeiro) da Chamada acima especificada e às Condições Gerais para Apoio Financeiro que regem a concessão dos recursos especificados abaixo:

**AUXÍLIO FINANCEIRO**

**Custeio:** R$ 20.000,00

**Capital:** R$ 9.200,00

**Valor Global:** R$ 29.200,00

**BOLSA DE LONGA DURAÇÃO**

**Modalidade:** Iniciação ao Extensionismo - IEX
**Duração:** 12 Meses
**Quantidade:** 2
**Modalidade:** Extensão no Pais - EXP
**Nível:** C
**Duração:** 12 Meses
**Quantidade:** 1

Tenho ciência:,

a) de que o prazo para utilização dos recursos financeiros começa a vigorar a partir da data da assinatura deste Termo de Aceitação, pelo período constante na Chamada correspondente, acrescido dos dias necessários para que a vigência final seja no último dia do respectivo mês de término; e

b) das disposições legais e procedimentos para a adequada utilização de recursos financeiros e a correta prestação de contas (Manual de Utilização de Recursos Financeiros e Prestação de Contas).

**1. DA CONCESSÃO:**

**1.1.** Ao aceitar o apoio financeiro, o BENEFICIÁRIO declara formalmente:

a) dedicar-se às atividades pertinentes à proposta aprovada;

b) observar o disposto nas Leis nº 8.666/93 e nº 10.973/04, nos Decretos nº 93.872/86 e nº 5.563/05 e na Lei nº 8.112/90, no que couber, bem como os demais instrumentos legais pertinentes;

c) conhecer o Protocolo de Cooperação Técnica firmado entre a instituição de execução do projeto/plano de trabalho e o CNPq, publicado no Diário Oficial da União;

d) conhecer e cumprir as exigências da Chamada à qual a proposta está relacionada, como também as normas do CNPq, ora em validade, relativas à modalidade de apoio financeiro aprovado, ciente que a eventual mudança dessas normas não afeta, altera ou incide sobre o presente documento, exceto quando proposta pelo CNPq e formalmente aceita pelo BENEFICIÁRIO;

e) possuir anuência formal da instituição de execução do projeto/plano de trabalho, seja sob a forma de vínculo empregatício ou funcional ou, na ausência deste, sob a forma de declaração de autoridade institucional competente, segundo modelo disponível na página do CNPq na Internet;

f) dispor das autorizações legais cabíveis de instituições como Instituto Brasileiro de Meio Ambiente e Recursos Naturais Renováveis - IBAMA, Fundação Nacional do Índio - FUNAI, Instituto Chico Mendes de Conservação da Biodiversidade - ICMBio, Jardim Botânico do Rio de Janeiro, além dos Comitês de Ética em Pesquisa - CEP, da Comissão Nacional de Ética em Pesquisa - CONEP, das Comissões de Ética em pesquisa com animais, da Comissão Nacional de Energia Nuclear - CNEN e outras, no caso em que a natureza do projeto, as exigir;

g) manter os documentos referidos nas alíneas "e" e "f" em seu poder até cinco anos após a aprovação final das contas do CNPq pelo Tribunal de Contas da União, não sendo necessária sua remessa ao CNPq;

h) ter ciência de que esta declaração é feita sob pena da incidência nos artigos 297-299 do Código Penal Brasileiro sobre a falsificação de documento público e falsidade ideológica, respectivamente; e

i) estar ciente que o prazo para utilização dos recursos financeiros começa a vigorar a partir da data da assinatura do Termo de Aceitação, pelo período constante na Chamada correspondente, devendo ser aplicados exclusivamente para a proposta aprovada.

**1.2.** O BENEFICIÁRIO compromete-se, ainda, a:

a) responsabilizar-se pela adequada implementação e aplicação dos recursos financeiros aprovados, atendendo aos aspectos normativos definidos para a(s) modalidade(s) concedida(s), podendo estar previsto apenas recursos de capital e custeio, como também recursos para bolsas;

b) utilizar os recursos financeiros em acordo com os critérios e procedimentos estabelecidos no Manual de Utilização de Recursos Financeiros e Prestação de Contas ;

c) assumir todas as obrigações legais decorrentes de contratações eventuais necessárias à consecução do objeto, não tendo tais contratações qualquer vínculo com o CNPq;

d) apresentar, nos prazos que lhe forem determinados, informações ou documentos referentes tanto ao desenvolvimento quanto à conclusão do projeto ou plano de trabalho aprovado;

e) se necessárias, propor alterações ao projeto/plano de trabalho, sujeitas à prévia análise e autorização do CNPq, e de entidade co-financiadora quando for o caso, desde que não se altere o objeto do projeto/plano de trabalho, e não implique remanejamento de despesas entre rubricas (capital para custeio e vice-versa);

f) permitir e facilitar ao CNPq o acesso aos locais de execução do projeto/plano de trabalho, o exame da documentação produzida e a vistoria dos bens adquiridos;

g) apresentar o relatório técnico final das atividades desenvolvidas em até 60 (sessenta) dias após o término da vigência do projeto/plano de trabalho, via Plataforma Carlos Chagas;

h) apresentar a prestação de contas financeira em até 60 (sessenta) dias após o término da vigência do projeto/plano de trabalho, em conformidade com o disposto no Manual de Utilização de Recursos Financeiros e Prestação de Contas, via Plataforma Carlos Chagas; e

i) se necessário, solicitar prorrogação de prazo de execução do projeto/plano de trabalho, via Plataforma Carlos Chagas, no prazo mínimo de 30 (trinta) dias antes do término da vigência.

**1.3.** É vedado

a) utilizar o recurso financeiro para fins distintos dos aprovados originalmente na proposta, sendo permitidas despesas exclusivamente com itens financiáveis estabelecidos nas normas de bolsas e auxílios individuais do CNPq, convênios e/ou Chamadas;

b) transferir a terceiros as obrigações assumidas sem prévia autorização do CNPq;

c) executar despesas em data anterior à vigência do benefício; e

d) efetuar pagamento em data posterior à vigência do benefício, salvo se expressamente autorizado pela autoridade competente do CNPq e desde que o fato gerador da despesa tenha ocorrido durante a vigência do Termo de Aceitação. Despesas realizadas fora do prazo de aplicação dos recursos serão glosadas.

## 2. DA GUARDA E DOAÇÃO DOS BENS

**2.1.** O BENEFICIÁRIO e a instituição de execução do projeto responderão pela manutenção do bem em perfeito estado de conservação e funcionamento.

**2.2.** Em caso de roubo, furto ou outro sinistro envolvendo o bem, o BENEFICIÁRIO ou a instituição de execução do projeto, após a adoção das medidas cabíveis, deverá comunicar imediatamente o fato ao CNPq, por escrito, juntamente com a justificativa e a prova de suas causas, anexando cópia autenticada da Ocorrência Policial, se for o caso.

**2.3.** É vedada a transferência dos bens para outro local ou estabelecimento, sem prévia e expressa autorização do CNPq. Todas as despesas decorrentes da transferência dos bens e os eventuais danos causados correrão por conta e risco do BENEFICIÁRIO e da instituição de execução do projeto.

**2.4.** A doação dos bens patrimoniais adquiridos com apoio financeiro do CNPq deverá ser efetuada conforme estabelecido em norma específica e com o disposto no Protocolo de Cooperação Técnica.

## 3. DA PROPRIEDADE INTELECTUAL / CRIAÇÃO PROTEGIDA

Caso os resultados do projeto ou o relatório em si venham a ter valor comercial ou possam levar ao desenvolvimento de um produto ou método envolvendo o estabelecimento de uma patente, a troca de informações e a reserva dos direitos, em cada caso, dar-se-ão de acordo com o estabelecido na Lei de Inovação, nº 10.973, de 2 de dezembro de 2004, regulamentada pelo Decreto nº 5.563, de 11 de outubro de 2005 e pela RN-013/2008.

## 4. DAS PUBLICAÇÕES E DIVULGAÇÃO

**4.1.** Trabalhos publicados e sua divulgação, sob qualquer forma de comunicação ou por qualquer veículo, de resultados obtidos com recursos do projeto, deverão, obrigatoriamente, no idioma da divulgação, fazer menção expressa ao apoio recebido do Conselho Nacional de Desenvolvimento Científico e Tecnológico - CNPq - Brasil, bem como mencionar quaisquer outras entidades/órgãos financiadores, especialmente aqueles que participaram no apoio do projeto em conjunto com o CNPq.

**4.2.** Material de divulgação de eventos, impressos em geral, publicações e a publicidade relativa a eles, de trabalhos e atividades apoiadas ou financiadas pelo CNPq, deverão trazer a logomarca deste em lugar visível, de fácil identificação em escala e tamanho proporcionais à área de leitura. Esclarecimentos a respeito e os padrões a observar devem ser objeto de consulta prévia junto à área de comunicação social do CNPq (comunicacao@cnpq.br).

**4.2.1.** Os mesmos materiais de divulgação de eventos, impressos em geral, publicações e a publicidade relativa a eles deverão trazer a logomarca de outras entidades/órgãos financiadores, em lugar visível, de fácil identificação, e em escala e tamanho proporcionais à área de leitura. (NR)

**4.3.** As ações publicitárias atinentes a propostas financiadas com recursos da União deverão observar rigorosamente as disposições contidas no § 1º do art. 37 da Constituição Federal, como também aquelas consignadas em Instrução Normativa da Secretaria de Comunicação de Governo e Gestão Estratégica da Presidência da República.

## 5. DA DESISTÊNCIA E SUSPENSÃO

**5.1.** Quando o BENEFICIÁRIO desistir da execução do projeto/plano de trabalho, antes do seu início, os recursos serão devolvidos ao CNPq, com justificativa plausível da desistência, no prazo de 30 (trinta) dias de seu recebimento. A não observância desse prazo implicará a correção do valor originalmente concedido, na forma da legislação aplicável aos débitos da Fazenda Nacional.

**5.2.** O BENEFICIÁRIO deverá comunicar formalmente ao CNPq qualquer descontinuidade do plano de trabalho ou do projeto de pesquisa, acompanhada da devida justificativa. No prazo de 30 (trinta) dias da comunicação da descontinuidade, deverão ser apresentados o relatório técnico e a prestação de contas, como também deverá ser devolvido ao CNPq eventual saldo financeiro. A não observância desse prazo implicará a correção do valor originalmente concedido, na forma da legislação aplicável aos débitos da Fazenda Nacional.

**5.3.** A liberação dos recursos do apoio financeiro ao projeto/plano de trabalho, bem como de quaisquer outros benefícios aprovados pelo CNPq, será suspensa quando ocorrer uma das seguintes impropriedades, constatada, inclusive, por procedimentos de fiscalização realizados pelo CNPq, Ministério da Ciência e Tecnologia - MCT, Secretaria Federal de Controle Interno - SFCI ou Tribunal de Contas da União - TCU:

a) não comprovação da utilização adequada da parcela anteriormente recebida, na forma da legislação pertinente, quando solicitada;

b) verificação de desvio de finalidade na utilização dos recursos ou dos bens patrimoniais adquiridos no projeto;

c) atrasos não justificados no cumprimento das etapas ou fases programadas no projeto/plano de trabalho; e

d) quando for descumprida qualquer condição deste instrumento.

**5.3.1.** A suspensão dos benefícios persistirá até a correção da causa verificada.

**5.4.** O BENEFICIÁRIO, cuja prestação de contas e relatório técnico final do projeto/plano de trabalho, com vigência expirada não forem aprovados, será considerado inadimplente e terá suspenso o pagamento de projetos/planos de trabalho, vigentes, bem como a concessão de novas modalidades de apoio, sem prejuízo de outras medidas julgadas necessárias pelo CNPq e previstas na lei.

**6. DAS DISPOSIÇÕES FINAIS**

**6.1.** As presentes condições gerais referem-se a proposta a ser financiada com recursos do CNPq. Se financiada com recursos de outras fontes, poderão prevalecer disposições específicas constantes em Chamadas, Convênios e outros regulamentos pertinentes.

**6.2.** O Termo de Aceitação só será válido na vigência do Protocolo de Cooperação Técnica firmado entre o CNPq e a instituição de execução do projeto/plano de trabalho, indicada pelo proponente na solicitação.

**6.3.** O apoio financeiro aprovado pelo CNPq não gera vínculo de qualquer natureza ou relação de trabalho, constituindo doação com encargos feita ao BENEFICIÁRIO.

**6.4.** O pessoal envolvido na execução do projeto/plano de trabalho, não possuirá vínculo de qualquer natureza com o CNPq e deste não poderá demandar quaisquer pagamentos, sendo estes de inteira responsabilidade do BENEFICIÁRIO/Instituição de execução do projeto/plano de trabalho, que o tiver empregado na sua execução.

**6.4.1.** Se eventualmente o CNPq for demandado pelo pessoal utilizado nos trabalhos, o BENEFICIÁRIO e a instituição de execução do projeto/plano de trabalho, o ressarcirão das despesas que em decorrência realizar, atualizadas monetariamente.

**6.5.** O processo somente será encerrado após as aprovações do relatório técnico final e da prestação de contas e desde que cumpridas todas as condições previstas neste instrumento e nas normas aplicáveis.

**6.6.** O descumprimento de qualquer condição constante deste instrumento e a inobservância de dispositivos legais aplicáveis implicará o encerramento imediato do apoio financeiro aprovado e obrigará o BENEFICIÁRIO a ressarcir integralmente o CNPq de todas as despesas realizadas, atualizadas nos termos da legislação, sem prejuízo da aplicação de penalidades cabíveis.

**6.6.1.** A recusa ou omissão do BENEFICIÁRIO, quanto ao ressarcimento de que trata este item, ensejará a consequente abertura de tomada de contas especial e a decorrente inscrição do BENEFICIÁRIO e do débito no Cadastro de Inadimplência Institucional - CADIN e do Tesouro Nacional.

**6.7.** O BENEFICIÁRIO reconhece que ao CNPq compete exercer a autoridade normativa de controle e fiscalização sobre a execução do projeto/plano de trabalho, bem como assumir ou transferir a responsabilidade pela mesma, no caso da paralisação ou de fato relevante que venha a ocorrer, de modo a evitar a descontinuidade das atividades.

**7. ACEITE**

Declaro ainda que li e aceitei integralmente os termos deste documento, comprometendo-me a cumpri-los fielmente, não podendo, em nenhuma hipótese, deles alegar desconhecimento.

*Termo de aceitação registrado eletronicamente por meio da internet junto ao CNPq, pelo agente receptor 10.0.2.21(srv257.cnpq.br), mediante uso de senha pessoal do Beneficiário em 27/11/2017, originário do número IP 200.130.33.73(200.130.33.73) e número de controle 2450017324500173:2435751824-1033730026.*

*Para visualizar este documento novamente ou o PDF assinado digitalmente, acesse: http://efomento.cnpq.br/efomento/termo?numeroAcesso=2786184626776925.*
